# Characteristics of Official Wheelchair Basketball Games in Hot and Temperate Conditions

**DOI:** 10.3390/ijerph19031250

**Published:** 2022-01-23

**Authors:** Fabian Grossmann, Joelle Leonie Flueck, Bart Roelands, Romain Meeusen, Barry Mason, Claudio Perret

**Affiliations:** 1 Swiss Paraplegic Centre, Sports Medicine, 6207 Nottwil, Switzerland; joelle.flueck@sportmedizin-nottwil.ch (J.L.F.); claudio.perret@paraplegie.ch (C.P.); 2 Human Physiology and Sports Physiotherapy Research Group, Vrije Universiteit Brussel, 1050 Brussels, Belgium; bart.roelands@vub.be (B.R.); romain.meeusen@vub.be (R.M.); 3 School of Sport, Exercise and Health Sciences, Loughborough University, Loughborough LE11 3TU, UK; barry.mason@gbwr.org.uk

**Keywords:** thermoregulation, heat stress, spinal cord injury, exercise

## Abstract

This study compared performance parameters of two wheelchair basketball games under hot (30.3 °C, 52% relative humidity) and temperate (21.6 °C, 30% relative humidity) environmental conditions and described the characteristics of wheelchair basketball. Eight wheelchair basketball players from two teams were monitored during two games using an indoor position tracking system. Total distance, mean- and peak-speed, playing-time, number of sprints, sprints per minute, heart rate and rate of perceived exertion were recorded. Additionally, athletes with a lesion level above and below T6 were compared. No measured parameter differed between the games. Across quarters (Q) mean velocity (m/s) (Q1: 1.01; Q2: 1.10; Q3: 1.18; Q4: 1.06; *p* < 0.001) and sprints per minute (Q1: 16; Q2: 14; Q3: 23; Q4: 14; *p* = 0.033) differed significantly, independent of the conditions. Descriptive statistics did not reveal differences between the groups with a lesion level below or above T6. In the present study, hot environmental conditions seemed not to have an impact on activity parameters of wheelchair basketball players. It was speculated that the game intensity and therefore metabolic heat production was too low; consequently, the athletes had a sufficient heat loss to prevent a decrease in performance during the play in hot conditions.

## 1. Introduction

Match and team performance in wheelchair basketball (WCB) are strongly influenced by the athletes’ mobility performance, which is determined by the interaction of the athletes’ physical performance and their wheelchair [1]. Physical performance is often quantified by means of oxygen uptake, heart rate (HR) and lactate production or drag force [2,3], whereas mobility performance is used to quantify the interaction of the athlete and his wheelchair in terms of acceleration, speed and covered distance [1,4]. Furthermore, activity profiles were used to describe the physical and energy demands of wheelchair team sports activities (i.e., WCB and wheelchair rugby) [5,6,7]. These demands, as well as the physical performance, might be influenced by external aspects such as the equipment (e.g., wheelchair, type of tires, tire pressure), floor type or environmental conditions (i.e., temperature, humidity) [8,9,10] and the status of personal heat acclimation as well [11]. WCB games are mostly played in air-conditioned sport facilities, which should provide uniform environmental conditions. However, not all facilities have an efficient air-conditioning system and therefore maintaining standard environmental conditions is difficult [12], and games in hot conditions (>28 °C) may be possible.

WCB is, among others, played by athletes with a traumatic spinal cord injury (SCI). As a consequence of the injury, the regulation of body core temperature is impaired among individuals with SCI [13]. This has been demonstrated to occur even during exercise in a cool ambient temperature, and it is further exacerbated during exercise in the heat [13]. The risk for heat-related illness and consequently a reduction in exercise performance is also elevated with increasing environmental temperature in athletes with SCI with a lesion level above T6 [13,14]. However, the effect of environmental heat stress on performance parameters and activity profiles has not been examined in an official game situation in wheelchair sports so far. Additionally, to date only a few studies published data on distance and speeds by WCB players during real gameplay [3,5,15,16,17] using different methodological approaches.

It has been shown that environmental conditions during exercise in able-bodied (AB) athletes trigger significant effects on the physiological responses during physical activity (i.e., increased HR, core temperature, sweat rate) [18]. Chmura, et al. [19] examined the performance of AB elite soccer players under different conditions. The most comfortable condition for physical exercise was at an air temperature below 22 °C and a relative humidity below 60% in this study. A temperature above 28 °C or 22 °C with a relative humidity > 60% led to decreased performance outcomes. Data from the same players showed that high environmental heat stress changed their activity patterns (i.e., reduced high-intensity activity, number of sprints) during matches [20]. In addition, research indicates that the challenges of hot environmental conditions in combination with exercise are complex and difficult to fully comprehend, as athletes are affected in different ways (e.g., heat exhaustion, muscle cramp, thermal discomfort) [21,22].

Hence, the present study aimed to compare performance outcomes of two official (friendly games, with official IWBF rules) WCB games, one under thermoneutral (TMP), a second under hot (HOT) conditions. It was hypothesized that due to the impaired thermoregulatory abilities of athletes with SCI, performance in HOT will be reduced (i.e., covered distance, number of sprints), and the activity patterns might change (i.e., less time in high-intensity but more in low-intensity speed zones).

## 2. Materials and Methods

### 2.1. Participants

**Figure 1:**
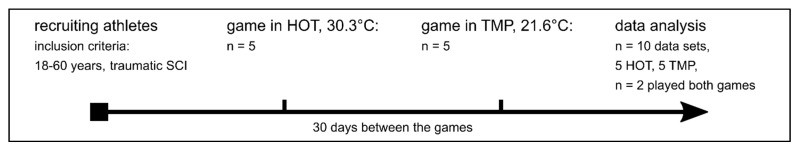
Study design, SCI = spinal cord injury, HOT = hot conditions, TMP = temperate conditions, n = number of participants.

In total, eight male WCB players volunteered to participate in the present investigation (Table 1). Two players played both games, the others in HOT or TMP, which resulted in five players per game. The detailed study design is presented in Figure 1. Inclusion criteria were age between 18 and 60 years, male, valid classification for WCB, member of a national team or in a team playing in Euro League and had a traumatic SCI. Athletes with an amputation, spina bifida or cerebral palsy were excluded. All participants were informed about the experimental protocol (orally and in writing), and all participants provided their written, informed consent prior to data collection. The study was approved by the local ethical committee (Ethikkommission Nordwest- und Zentralschweiz, Basel, Switzerland). All procedures were conducted according to the Helsinki Declaration [23].

### 2.2. Procedure

Data were collected from two official WCB games (friendly games with official IWBF rules), played over four quarters of 10 min (effective time) on two separate days. Environmental conditions in HOT were 30.3 °C and 52% relative humidity and in TMP 21.6 °C and 30% relative humidity (Irox JB913R, Electronic Thermo-Hygrometer, OS Technology AG, Guemligen, Switzerland). One hour before the game, athletes were again informed about the detailed procedure and equipped with a HR monitor (Acentas Herzfrequenz-Monitoring Team System, Acentas GmbH, Hoergertshausen, Germany). A lightweight tag for position tracking was mounted on the wheelchair frame as close as possible to the pivot and point of rotation. Athletes performed their usual, individual pre-game warm-up with a duration of 15 min at a perceived exertion between 10 and 12 on the Borg-scale [25]. Data collection began with the start of the match clock and was paused during time-outs, breaks between quarters and when a player was on the bench. Immediately after the game, participants were asked to rate their perceived exertion on a Borg-scale from 6 to 20 [25]. Heart rate was measured with a sample rate of 1 Hz. The recording was started with the beginning of the game. Playing time (PT) for each player was assessed by a commercially available digital stopwatch (Delta, Sport-Thieme AG, St. Gallen, Switzerland).

### 2.3. Equipment/Location/Instruments

Participants played in their own basketball-specific wheelchairs, which were weighed. Wheel diameter, camber angle were determined and type of tire and pressures were noted individually to guarantee uniformity in both games. Games were played in an indoor sports complex with a hardwood flooring, typical for indoor wheelchair team court sports. WCB court has the same dimensions as the court for AB basketball players (28 m × 15 m). The sport hall was heated naturally. Environmental temperature and relative humidity were measured throughout (values were written down every 10th minute; mean was calculated at the end) with a thermo- and hygrometer and were constant during the game.

The ITS (XLOCATE, Axiamo, Biel, Switzerland) is a position tracking system based on ultra-wideband signals. Six wireless reference antennas were located around the playing field, one in each corner and two parallel to the halfway line to have an optimal court coverage. The tags mounted on wheelchairs emitted ultra-wideband signals and were tracked by means of time difference of arrival and triangulation. Tags were sampled at 20 Hz with a precision of ± 20 cm. The fully mobile configuration was based on wireless technologies and battery-powered devices. The tags came in a small form factor and were lightweight (size = 50 × 80 × 9 mm, weight = 30 g). Raw data files were exported and filtered using a 3-pass sliding-average filter with a window width proportional to the tag frequency [26]. Afterwards, total distance in meters, mean velocity (V_mean_, m·s^−1^), peak velocity (V_peak_, m·s^−1^), number of sprints, number of sprints per min, PT and percentage of time spent in six different speed zones (Z1: 0.0–0.49 m/s, Z2: 0.5–0.99 m/s, Z3: 1.0–1.49 m/s, Z4: 1.5–1.99 m/s, Z5: 2.0–2.49 m/s, Z6: >2.5 m/s) were calculated. A sprint was defined when velocity was larger than two meters per second on a duration longer than two seconds. The Z were adopted from wheelchair rugby [27].

### 2.4. Statistics

Data were analyzed using the software R (R Foundation for Statistical Computing version 3.6.0; Vienna, Austria). After checking all measured parameters for normality using Shapiro–Wilk’s tests, median and interquartile range (IQR) were calculated for all parameters. Due to nonparametric data, mean differences between both games were explored using Wilcoxon’s signed rank tests. To detect significant changes over the course of the four quarters the Brunner–Munzel Test [28] was used. Wilcoxon’s signed-rank test with Bonferroni *p*-level adjustment was used as post hoc test. Sample size estimation using G*Power software (v.3.1.9.2, Franz Faul, Kiel, Germany) [29] with the assumption of a significant difference in V_mean_ of 0.15 m/s ± 0.07 m/s revealed a power of >80% with 5 study participants per game. The results were evaluated within a 95% confidence interval and at a statistical significance level of *p* < 0.05. Additionally, data were split up to above and below lesion level T6 and are presented in median.

## 3. Results

### 3.1. HOT vs. TMP

Activity patterns did not differ between HOT and TMP (Table 2). Time spent in different Z did not differ between the two games (Table 3). Descriptive statistics did reveal remarkable differences in covered distance, total sprints, V_peak_ and PT between players with a lesion level above and below T6 (Table 4 and Table 5).

### 3.2. HOT and TMP (Overall Data)

**Figure 2:**
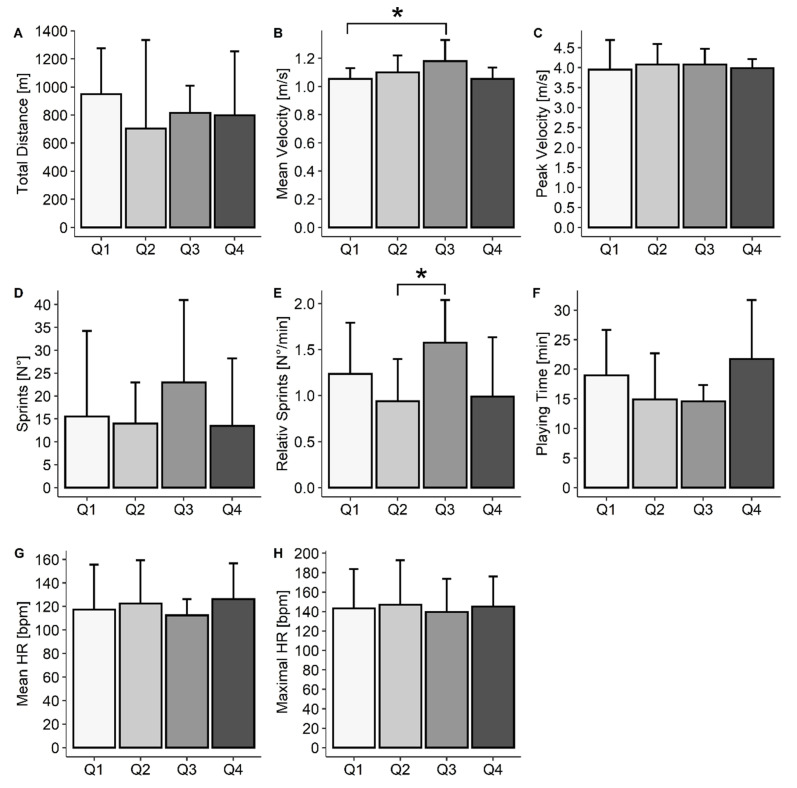
Overall data presented in median ± interquartile range (IQR), total distance (**A**), mean velocity (**B**), peak velocity (**C**), number of sprints (**D**), sprints per minute (**E**), playing time (**F**), mean heart rate (**G**), maximal heart rate (**H**) during each quarter (Q), * Significant difference ((**B**), *p* = 0.0312; (**E**), *p* = 0.063).

Across the quarters, V_mean_ (*p* < 0.001) and number of sprints per minute (*p* = 0.033) differed significantly. In quarter 1 (Q1), V_mean_ was significantly lower compared with quarter 3 (Q3) (*p* = 0.031), and the number of sprints per minute was significantly smaller in quarter 2 (Q2) compared with Q3 (*p* = 0.042). Total distance (*p* = 0.766), V_peak_ (*p* = 0.776), PT (*p* = 0.294), number of sprints (*p* = 0.801), HR_max_ (*p* = 0.605) and HR_mean_ (*p* = 0.355) did not differ across quarters (Figure 2).

### 3.3. Speed Zones

**Figure 3:**
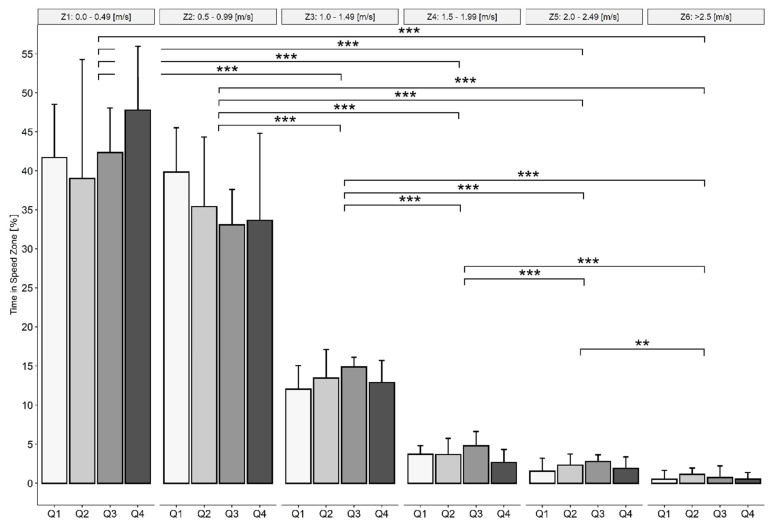
Percentage of time spent in different speed zones (Z), median ± interquartile range (IQR), *** significantly different from other zones (*p* < 0.001), ** significantly different from other speed zone (*p* = 0.002).

Percentage of time spent in six different speed zones (Z1–Z6) differed significantly across the zones (*p* < 0.001). Athletes spent significantly more time in Z1 compared with Z3–Z6 (for all differences: *p* < 0.001), significantly more time in Z2 compared with Z3–Z6 (for all differences: *p* < 0.001), significantly more time in Z3 compared with Z4–Z6 (for all differences: *p <* 0.001), significantly more time in Z4 compared with Z5 and Z6 (for both differences, *p* < 0.001) and significantly more time in Z5 compared with Z6 (*p* = 0.002) (Figure 3).

## 4. Discussion

The present study was the first to analyze the effect of environmental heat stress on performance parameters and activity profiles in an official WCB game and to compare these parameters with an official game under temperate conditions. The results showed that playing WCB under hot or temperate conditions seemed not to affect the performance of WCB players in the present study.

### 4.1. Comparison of HOT and TMP

The current study demonstrates that activity parameters such as total distance, V_mean_, V_peak_, number of sprints, number of sprints per min, PT and time in different speed zones, as well as HR and rating of perceived exertion, did not significantly differ between the games. Because this is the only study in wheelchair team sports so far, the comparison of hot and temperate conditions on the performance of individuals with SCI with other data is not possible.

In AB, it was observed that hot environmental conditions seem to influence the activity patterns (i.e., reduced high-intensity activity, total distance) [20]. As a result of the loss of vasomotor control and the absence of sweating response below the lesion level in individuals with a SCI (the sympathetic innervation of the eccrine sweat glands exits the spinal cord at T1-L2) [30,31], it has to be noted that the impairment of the thermal and thermoregulatory responses depends on the level of the lesion and the completeness of the spinal rupture [13]. Therefore, it can be expected that the performance of players with a low-level lesion would not be affected by the same amount as in cases of athletes with a high-level lesion. Nevertheless, if an AB game is affected by reason of heat strain, at least the same would be expected for an SCI game, but this could not be observed in the present study. Players spent over 70% of the time in the lowest Z1 and Z2. It has been shown in the past that low intensity exercise is related to a small metabolic heat production [32]. Therefore, it is suggested that the stored heat in the body was not high enough to get overheated and affect performance. Thus, the ability to lose heat was still adequate and therefore performance was not influenced negatively. It can be argued that low lesion level players with a lesion below T6 are able to thermoregulate their body similar to AB. Players with a high lesion level (i.e., above T6) are associated with considerably worse thermoregulation [13]. Hence, it makes sense to take a closer look at players with a lesion level above and below T6 (Table 4 and Table 5). The numbers reveal some remarkable differences but without a concrete direction that would allow a conclusion. Due to the small number of participants and large differences between the single subject, no clear pattern can be shown. Again, the above-mentioned low metabolic heat production can be used as an argument as well. The low-intensity during the game did not trigger a sufficient heat production to provoke differences between these two groups.

### 4.2. Performance Characteristics during WCB Game

Since no difference was found between the two conditions, the two games were analyzed together to provide data on the characteristics of WCB. It seems noteworthy that this was the first time that data were provided separated in quarters. Whereas total distance, V_peak_, PT, number of sprints, maximal and mean HR did not differ across quarters, number of sprints per minute and V_mean_ significantly differed across the quarters (Figure 1). Despite these differences, there was no clear pattern to determine which specific quarter revealed to be the one with the strongest performance (i.e., highest V_mean_, highest number of sprints, highest mean HR).

The descriptive WCB data provide quantification of activity and performance during a game. Total distance, V_mean_, V_peak_, number of sprints, PT, time in different speed zones, rate of perceived exertion and HR of two games were described. The finding differs in several parameters from the existing literature. Coutts [3] (1.98 m·s^−1^), Sporner et al. [5] (1.48 m·s^−1^) and Mason et al. [15] (1.56 m·s^−1^) presented notably higher V_mean_ values than presented in the current work (1.10 m·s^−1^). Coutts [3] found similar values for V_peak_ (4.04 m·s^−1^), whereas Mason et al. [15] found slightly higher values (4.69 m·s^−1^). Comparing total distance, each study had completely different findings. The large differences can mainly be explained by the different methods of collecting data. Coutts [3] only analyzed a portion of 6 min of an exhibition game, and values were collected with a magnetic reed switch mounted together with a laptop computer on the wheelchair, at which it is unclear when data collection was paused. Therefore, it is unclear whether data on moving during timeouts or calls by the referee were collected. Mason et al. [15] used a radio frequency-based indoor tracking system [26] and paused the collection during extended stoppages (e.g., timeouts, equipment calls). In the present study, the collection was only paused during timeouts. These differences in tracking modalities can lead to a difference in collected distance and thus a wrong calculation of V_mean_. Another aspect is the PT. Mason et al. [15] indicated a mean PT of 20:23 min, while in the present study 58:00 min were recorded. It is suggested that the longer the PT, the more weight is given to interruptions (e.g., calls by the referee) in the calculation of the various values.

### 4.3. Speed Zones

Taking a closer look at the distribution of the percentage of PT in different Z unveils that 40% of the PT the players was spent in Z1, around 35% in Z2 and 13% in Z3. The athletes spent in total more than 80% of the PT between standstill and a velocity of 1.5 m·s^−1^. This is nearly 25% more than Mason et al. [15] have observed. It is suggested that less participation time can lead to an increased intensity, and therefore the recorded values such as V_mean_ must be higher. This matches with the measured HR values. Whereas Mason et al. [15] and Croft et al. [33] described a mean HR of around 155 beats per minute, the present study reported a value of 118 beat per minute, which is associated with a much lower exercise intensity. Since some athletes with SCI may have a disturbed HR regulation [34], and in other studies often other disabled athletes were included without SCI, this comparison has to be taken with caution.

When comparing Z with other wheelchair court sports, the level of performance matches with that of rugby (i.e., similar V_mean_) [6]. Nevertheless, this does not match with the earlier investigation that reveals WCB as the sport with the highest performance (i.e., lower time spent in low-intensity zones) and rugby the one with the lowest [35]. It should be noted that the difference might be due to the use of different tracking systems, and therefore the findings by van der Slikke et al. [4] (activity profile of WCB) should be given more weight, as the different sports were measured with the same tracking tool.

### 4.4. Limitations

The recruitment of an appropriate number of participants is difficult since athletes with SCI represent a small group in the general population. The few participants included in this study, different participants under different conditions and a combination of athletes with a lesion level above and below T6 leads to a low power and therewith, the chance of detecting a true error. The chance that significant results reflect a true effect are decreased, and therefore an overestimation of the effect sizes is common [36]. Therefore, there needs to be a careful balance between not overrating significant findings and devaluing insignificant results. Another problematic issue is that each game of WCB has its own character. The two teams competing (i.e., different opponents), the team tactics, number of substitutions, field position of the players, the calls of the referees, or numbers of interruptions (i.e., timeouts, equipment calls) are important factors, which make a comparison of two games very difficult. Grouping athletes based on the lesion level also means selecting athletes with different playing-position and therefore different roles on court, which can lead to completely different performance parameters [1]. Due to the lack of prior research on the topic, the findings cannot be verified. Thus, field tests, which mimic WCB games, are necessary to conduct “games” under standardized conditions to compare physiological parameters between different conditions or between different lesion levels.

Comparing the merged data of both games with the existing literature [3,5,15], remarkable differences in V_mean_ or PT are depicted. It is suggested that this disparity can also be a methodological issue. First, V_mean_ depends on the way it was calculated (i.e., covered distance divided by the overall time or active game time). Second, to collect the data, different tracking systems were used, which may not record data in the same way (i.e., different sensors, different sensor placements at the wheelchair, different recording protocols). Therefore, a guideline for the use of tracking systems, with information about the data collection process (i.e., when has the data collection to be paused) and the filtering of the data would be very useful. This would make the different systems more comparable and therefore the outcome data as well. Additionally, the development of a field test, which mimics the game as closely as possible, is essential and should be a further direction of research.

### 4.5. Practical Applications

Even if the generated data during both WCB games did not provide significant differences, the findings suggest that hot environmental conditions in WCB do not seem to be a major problem for individuals with a SCI. Nevertheless, it has to be considered that players with high-level lesions have impaired thermoregulatory functions and need more attention in future research. The characteristics of WCB change by nature from game to game. Therefore, for a better comparability of two environmental conditions, it is, on the one hand recommended to develop a field test that mimics the real games, and, on the other hand to investigate the variance in game performance as seen in AB soccer [37]. Only then would it be possible to make any suggestions and assignments on how to train and play under hot conditions. Finally, the market for tracking devices is growing rapidly and the tracking of indoor wheelchair sports activities is becoming easier and easier. However, the more tools that are available, the more difficult the comparison becomes. Thus, it is important to provide guidelines that point out the important determinants of collecting data with a tracking device.

## 5. Conclusions

In conclusion, this study demonstrated that a competitive WCB game in HOT seems not to have a different outcome regarding activity parameters compared with a game in TMP. Our finding suggests that overall intensity during these specific games was too low, and thus it is speculated that the produced metabolic heat was too low. Consequently, the athletes had sufficient heat loss, and performance was not degraded. The current setup included too many variables that could affect the outcome. Therefore, for future research, it would be a good to reduce the number of variables by first testing in a more standardized match such as a field test with maximal performance. If that reveals differences, a next step would be to test it in a more ecologically valid match setting.

## Figures and Tables

**Table 1:** Participants’ characteristics.

Subject	Age (years)	Lesion Level	Classification	Weight (kg)	Game Played
1	29.3	L1	4.0	82.8	HOT
2	39.7	C5	2.5	94.6	HOT, TMP
3	46.3	T5	1.0	78.6	HOT, TMP
4	40.2	T4	1.0	83.0	HOT
5	29.6	T10	3.0	70	HOT
6	42.4	L1	4.0	110.6	TMP
7	59.7	T10	2.0	111.6	TMP
8	20.0	T8	1.0	74.2	TMP
Median, IQR	40.0, 13.2	-	-	82.9, 16.0	-

IQR = interquartile range; HOT = game in hot conditions; TMP = game in temperate conditions; Classification refers to IWBF Official Player Classification [24].

**Table 2:** Activity patterns of two wheelchair basketball games, one in hot and one in temperate environmental conditions.

	HOT		TMP		HOT and TMP Merged		Difference HOT vs. TMP
Measured Parameter	Median	IQR	Median	IQR	Median	IQR	*p*
Total distance [m]	3286.7	1906.8	3477.6	1458.2	3332.0	1853.6	0.31
V_mean_ [m·s^−1^]	1.12	0.1	1.07	0.1	1.1	0.1	0.54
V_peak_ [m·s^−1^]	4.15	0.91	4.12	0.35	4.14	0.5	0.69
Number of sprints	89	61	68	30	68	53	1.00
Relative sprints [sprint/min]	1.5	0.4	1.0	0.3	1.2	0.4	0.22
PT [min]	54.8	27.0	69.4	35.6	58.0	33.0	0.31
HR_mean_ [bpm]	122	22	116	7	118	22	0.42
HR_max_ [bpm]	173	7	144	5	156	32	0.42
RPE [Borg]	15	2	15	1	15	0.75	0.21

HOT = game in hot conditions; TMP = game in temperate conditions; V_mean_ = mean velocity; V_peak_ = peak velocity; PT = playing time; HR_max_ = maximal heart rate; HR_mean_ = mean heart rate; IQR = inter quartile range; *p* = *p*-value.

**Table 3:** Percentage of time spent in different speed zones of two wheelchair basketball games, one in hot and one in temperate environmental conditions.

	HOT		TMP		HOT and TMP Merged		Difference HOT vs. TMP
Measured Parameter	Median	IQR	Median	IQR	Median	IQR	*p*
Z1 0.0–0.49 m·s^−1^ [%]	39.4	8.5	48.2	6.8	43.7	8.2	0.09
Z2 0.5–0.99 m·s^−1^ [%]	40.7	5.2	34.4	7.5	37.2	6.9	0.15
Z3 1.0–1.49 m·s^−1^ [%]	12.2	1.9	13.1	1.7	12.3	1.5	0.84
Z4 1.5–1.99 m·s^−1^ [%]	4.2	1.1	3.5	5.2	3.7	0.8	0.31
Z5 2.0–2.49 m·s^−1^ [%]	2.4	0.6	1.7	0.5	2.0	0.9	0.31
Z6 > 2.5 m·s^−1^ [%]	0.8	1.2	0.5	0.6	0.6	0.9	0.31

HOT = game in hot conditions; TMP = game in temperate conditions; IQR = interquartile range; *p* = *p*-value.

**Table 4:** Comparison of players with a lesion level above and below T6.

	HOT		TMP		Differences between Lesion Levels	
Measured Parameter	Above T6	Below T6	Above T6	Below T6	HOT	TMP
Total distance [m]	1470	3368	3582	2057	−1898	1525
V_mean_ [m·s^−1^]	1.11	1.22	1.04	1.12	−0.11	−0.08
V_peak_ [m·s^−1^]	3.9	4.95	4.28	4.12	−1.05	0.16
Number of sprints	30	92	68	38	−62	30
relative sprint [sprint/min]	1.2	1.8	0.9	1.3	−0.6	−0.4
PT [min]	34.3	58.0	73.8	35.6	−23.7	38.2
HR_mean_ [bpm]	118	147	127	116	−29	11
HR_max_ [bpm]	167	179	152	144	−12	8
RPE [Borg]	13	16	16	15	−3	1

HOT = game in hot conditions; TMP = game in temperate conditions; V_mean_ = mean velocity; V_peak_ = peak velocity; PT = playing time; HR_max_ = maximal heart rate; HR_mean_ = mean heart rate.

**Table 5:** Comparison of time spent in different speed zones of players with a lesion level above and below T6.

	HOT		TMP		Difference between Lesion Levels	
Measured Parameter	Above T6	Below T6	Above T6	Below T6	HOT	TMP
Z1 0.0–0.49 m·s^−1^ [%]	39.4	39.4	44.6	48.2	−5.2	−8.8
Z2 0.5–0.99 m·s^−1^ [%]	37.6	40.7	36.5	31.1	1.1	9.6
Z3 1.0–1.49 m·s^−1^ [%]	14.1	12.1	13.3	13.1	0.8	−1
Z4 1.5–1.99 m·s^−1^ [%]	3.9	4.7	3.1	3.9	0.8	0.8
Z5 2.0–2.49 m·s^−1^ [%]	2.7	2.1	1.5	1.9	1.2	0.2
Z6 > 2.5 m·s^−1^ [%]	2.0	0.5	0.6	0.5	1.4	0

HOT = game in hot conditions; TMP = game in temperate conditions; Z = speed zone.

## Data Availability

The datasets used and analyzed during the current study are available from the corresponding author on reasonable request.
